# A Guide to Therapeutics and Materia Medica

**Published:** 1878-04

**Authors:** 


*A Guide to Therapeutics and Materia Medica.* By ROBERT FARQUHARSON, M. D. Edin. F., R. C. P. London. Lecturer on Materia Medica at St. Mary's Hospital Medical School, etc. Enlarged and Adapted to the U. S. Pharmacopœia by FRANK WOODBURY, M. D. Member of the Academy of Natural Sciences, Phila., etc. Phildelphia: Henry C. Lea. 1877, Buffalo: T. H. Butler. pp. 410.

A well considered attempt is made in this volume to render the abstruseness of therapeutics more rapidly comprehensible to the learner. Its brevity and clear unequivocal statements, comparison upon the same page of the physiological and therapeutical actions of medicines, tabulation of the different preparations of each drug and medium dose, antidotes, and how to use them, arranged in connection with every possible poison mentioned, local and constitutional effects under distinct heads, all aid in securing the time and labor of the student. For this end also the complete primary list of the U. S. Pharmacopœia is alphabetically placed, so that instead of first searching through an index, needed information can be found at once if found at all. Remarks on certain classes of remedies as diaphoretics, astringents, &c. are sufficiently full and reliable. So are rules for prescribing. Dr. Woodbury's adaptation of the contents of the book to the wants of American students is neatly and well done. A volume might be written upon each of these medical agents. That the author has succeeded in stating so many facts in so few words is but one of the many distinctive features of excellence in the work.

